# Molecular Hydrogen Reduces Electromagnetic Pulse-Induced Male Rat Reproductive System Damage in a Rodent Model

**DOI:** 10.1155/2022/3469474

**Published:** 2022-09-25

**Authors:** Long Ma, Wei Hao, Wen-Bo Feng, Lu-Cheng Cao, Li-Na Qin, Yao Wang, Ming-Hua Liu, Neng-Zhuang Wang, Fu Gao, Jia-Ming Guo, Hui Du, Hong-Li Yan

**Affiliations:** ^1^Department of Radiation Medicine, College of Naval Medicine, Naval Medical University, Shanghai 200433, China; ^2^Department of Clinical Laboratory, Beidaihe Rehabilitation and Recuperation Center of PLA, Qinhuangdao 066100, China; ^3^Center of Reproductive Medicine, First Affiliated Hospital, Naval Medical University, Shanghai 200433, China; ^4^Department of Endocrinology, First Affiliated Hospital, Naval Medical University, Shanghai 200433, China; ^5^Administration Office for Undergraduates, Naval Medical University, Shanghai 200433, China

## Abstract

Infertility has got to be a broadly concerned social issue these days, in which the malefactor cannot be overlooked. Numerous studies have shown that electromagnetic pulse (EMP) radiation may have seriously damaging effects on reproductive health, through nonthermal effects and oxidative stress. Molecular hydrogen, a selective hydroxyl radical scavenger, explains the protective effects against many diseases closely associated with oxidative damage, such as ionizing radiation (IR). We sought to characterize the beneficial effects of molecular hydrogen on the male reproductive system in a rodent EMP exposure model. The 8-week-old male Sprague-Dawley rats were exposed to EMP (peak intensity 1000 kV/m, pulse edge 20 ns, pulse width 200 ns, 1 Hz, and 200 pulses), with or without hydrogen-rich water. The pathological structure of the testis, the rate of apoptosis of the testis, the serum testosterone level, the sperm parameters, and the activity of the antioxidant enzymes of the testis were measured. Then, transcriptomic and untargeted metabolomic analyses were applied to uncover the underlying mechanism. Exposure to EMP increased testicular apoptosis rate and apoptosis protein level, decreased sperm viability and motility, decreased serum testosterone levels, and diminished testicular antioxidant capacity. Molecular hydrogen-alleviated damage decreased the testicular apoptosis rate and apoptosis protein level, increased sperm motility, increased serum testosterone levels, and improved antioxidative capacity. Omics results showed that molecular hydrogen has a strong influence on metabolic pathways, and EMP affects mainly oxidative phosphorylation, TNF signaling pathways, and cytokine-receptor interactions. The mechanism of molecular hydrogen's effect may be related to the reversal of some metabolite levels. These observations warrant molecular hydrogen as an innovative approach for potential protection against EMP.

## 1. Introduction

Male reproductive problems have become quite common worldwide [1]. It should be noted that infertility affects an estimated 15% of couples worldwide and male reproductive problems are considered to account for 50% of all cases [2]. Male reproductive problems have become a worldwide concern.

With the development of electronic technology, electromagnetic radiation (EMR) has been widely used in various fields including communication, industry, and especially military areas. Thus, EMR has been recognized as one of the fast-growing environmental pollution sources, whose impact on human health has attracted much more public attention than before [3]. Modern people have been exposed to quite complex EMR environments since the day they were born, for example, the frequency of which ranges from extremely low frequency, power frequency, radiofrequency, to microwave, not to mention that the most common EMR exposed scenes are a composition of several kinds of continuous and/or various pulsed electromagnetic waves. Spermatogenesis is highly susceptible to exposure to the external environment, including EMR [4]. Evidence from epidemiological and experimental studies has generated a consensus that overexposure to EMR could damage reproductive health issues, including destruction of testicular tissue microstructure, decreased sperm concentration, reduced sperm motility, decreased serum testosterone levels, sexual dysfunction, abnormal embryonic development, congenital deformity, perinatal death, and abnormal development in utero [5–9].

The damage of biological tissue caused by EMR is mainly through thermal effect and nonthermal effect, and the latter is the focus of recent research. Studies have shown that electromagnetic radiation produces nonthermal effects primarily by altering the redox system of biological tissues, such as increasing reactive oxygen species (ROS) levels, decreasing antioxidant enzyme activity, and increasing levels of lipid oxidation products [10, 11]. Increasing in level of ROS results in damage to DNA, destruction in the endothelium of seminiferous tubule, and apoptosis in testicular germ cell [12, 13].

Given the negative effects of EMR on the human body, especially the reproductive system, it is extremely urgent to find safe and effective EMR protectants. Present research on EMR protectants is mainly focused on two aspects. One is the nonenzymic antioxidants in organisms, such as tocopherol and melatonin [14–16]. The other kind is a natural antioxidant or its extracts, such as Arborescens aloe juice and the selenium rich Cordyceps fungi [9, 17]. However, the former often exerts extensive functions, and excessive intake will cause body dysfunction. And the latter need to be dissolved in solvents and often have certain toxicity. Collectively, neither of the current EMR protectants is suitable enough and new candidates with low toxicity, high effectiveness, and fine bioavailability are urgently needed to be exploited.

Molecular hydrogen is the smallest molecule in its natural state. It is colorless, nontoxic, and permeable to biofilm. Molecular hydrogen has long been used as “inert gas” in biomedicine due to its lack of metabolizable enzymes. In 2007, Ohsawa et al. reported that molecular hydrogen can effectively alleviate oxidative damage by selectively scavenging hydroxyl radicals and nitro peroxide anions [18], which opened a surge of hydrogen biological studies in the following years. Until now, the antioxidant capacity of molecular hydrogen has been verified in many fields. In our previous work, we have systematically explicated the protective effects of hydrogen against IR and unveiled parts of the mechanism [19–22]. We found that its excellent performance on radioprotection is relevant to its bioactivity of antioxidant, anti-inflammatory, antiapoptosis, and energy metabolism regulation [23–25]. Thus, as EMR and IR share some common routines that cause orgasm damage, such as producing excessive reactive oxygen/nitrogen species, we assume that molecular hydrogen could also prevent EMR injury. Here, we investigate whether molecular hydrogen could alleviate the male reproductive system damage following exposure to EMP exposure and the possible mechanisms.

## 2. Materials and Methods

### 2.1. Ethical Statement

The experimental study was reviewed and approved by the Naval Medical University Institutional Animal Care and Use Committee (Shanghai, China, ethical code: CHREC2019-2). All living conditions and protocols were approved by the Naval Medical University Institutional Animal Care and Use Committee following the Guide for the Care and Use of Laboratory Animals published by the US NIH (publication No. 96-01).

### 2.2. Animals

Wild-type male Sprague-Dawley (SD) rats (8 weeks old, weighing 200–220 g) were received from the Shanghai Laboratory Animal Center of the Chinese Academy of Science and kept at 23° C to 25° C with a 12 h light/dark cycle. Before the experiment, rats were housed for one week to adapt to the new environment.

Seventy-five rats were randomly divided into three groups (25 rats per group): the sham group, the EMP group, and the EMP + H_2_ group. Rats in each group were sacrificed at 5 time points: 3 h, day 1, day 3, day 7, and day 10 after EMP exposure, 5 rats per time point.

For transcriptomic and metabolomic analyses, 32 rats were randomly assigned to four groups: the sham group, the H_2_ group, the EMP group, and the EMP + H_2_ group. Each group encompassed 8 rats. These rats were sacrificed 1 day after EMP exposure.

### 2.3. EMP Exposure System

**Figure 1:**
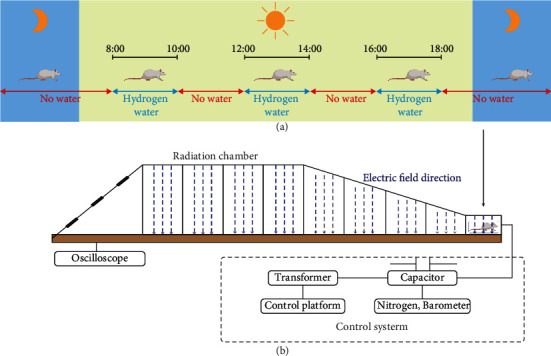
EMP exposure and molecular hydrogen administration. (a) Schematic diagram of the molecular hydrogen administration. The EMP + H_2_ group and the H_2_ group received hydrogen-rich water at 8-10, 12-14, and 16-18 o'clock, while the sham group and the EMP group received ordinary drinking water. (b) Schematic diagram of EMP exposure process. The pulse width selected in this experiment is fixed at 200 ns, and the rising edge is 20 ns. Each rat was exposed to 200 pulses with an electromagnetic field intensity of 1000 kV/m.

The EMP exposure system used in this study was designed and built at the Department of Biophysics, East China Normal University, Shanghai, China. The system is shown in Figure 1(b), which is composed of a voltage control system (control platform, booster transformer, and pulse capacitor), an air pressure control system (high-purity nitrogen gas, air valve, and barometer), a radiation chamber, and a pulse detection system (voltage divider, coaxial cable, and oscilloscope). Before radiation exposure, the high purity nitrogen gas in the pulse capacitor is controlled by the air pressure control valve and air pressure meter, and the voltage between the two plates of capacitance is increased by a booster transformer. When the voltage reaches a certain value, the nitrogen gas is broken down, and an electrical pulse is generated between the metal lead plates connected to the capacitance, which propagates along with the radiation chamber to the end [26]. The EMP is attenuated by resistance to prevent reflection formation. Based on our preliminary experimental results, 1000 kV/m, the maximum voltage of this exposure system, was adopted to investigate the acute exposure injury induced by EMP. And an oscilloscope connected to the sampling resistor of the irradiation chamber was used to detect the waveform (pulse edge 20 ns, pulse width 200 ns, 1 Hz, and 200 pulses).

In this study, the molecular hydrogen administration method was based on free drinking of saturated hydrogen-rich water (Figure 1(a)). Before the formal experiment, to train rats to drink water, rats were fed intermittent drinking water for 1 week, and water was only administered at 8-10, 12-14, and 16-18 o'clock every day. Three days before EMP radiation, the water fed to the rats of the EMP + H_2_ group was replaced with freshly prepared hydrogen-rich water, which was maintained until the animal sacrifice day. The concentration of hydrogen in hydrogen-rich water is shown in Figure S1.

### 2.4. Determination of Biometric Parameters

Rats were weighed before anesthetizing with isoflurane at the aforementioned time points. After anesthesia, two testicles from both sides of each animal were dissected, weighed, and fixed or frozen with liquid nitrogen as soon. Subsequently, the testicular index was calculated using the following formula [27]:

(1) $$\begin{array}{c} \text{Testis index}=\frac{\text{testis weight}\left(g\right)}{\text{body weight }\left(g\right)}\times 1000‰ \end{array}$$

### 2.5. Measurement of Seminiferous Tubule Diameter and Johnsen Score

The right testis of each rat was dissected, fixed with 4% paraformaldehyde for at least 24 hours, and then dehydrated with a graded ethanol series. The sample was then embedded in paraffin and cut into 4*-μ*m-thick slices for further staining analysis. Hematoxylin and eosin (HE) staining was then used to evaluate the histopathological alteration of the microstructure. According to the method of Miao et al. [9], 40 seminal tubules were randomly selected from each rat, and the diameters of the seminiferous tubules were measured across the minor and major axes. The Johnsen score [28] was used to analyze the histopathological characteristics of the testis, and 80 seminal tubules were randomly selected from each rat.

### 2.6. TUNEL Staining

Spermatogenic cell apoptosis was detected using terminal deoxynucleotidyl transferase-mediated fluorescent labeling of the nick end (TUNEL), according to the manufacturer's protocol (Servicebio, Wuhan, China). Dehydrated slices were treated with protease K for 20 min and washed with phosphate buffered saline (PBS) twice. The slices were treated with membrane breaking solution at room temperature for 20 min and washed twice with PBS. Then, equilibration solution was added to cover the samples and incubated at room temperature for 30 min, followed by TUNEL reaction mixture and incubation at 7°C for 2 h. After washing with PBS for 4 times, the slides were immersed in a dyeing cylinder containing 1 *μ*g/mL 4,6-diamidino-2-phenylindole (DAPI) solution and dyed for 8 min. Digital images were taken from each stained tissue section (Pannoramic DESK, P-MIDI, P250, Hungary). The percentage of TUNEL-positive cells was calculated using the ImageJ analytical software.

### 2.7. Western Blot Assay

The protein was extracted by using M-PER mammalian protein extraction reagent (Thermo Fisher Scientific) according to the manufacturer's instructions. After blocking for 1 hour at room temperature, the PVDF membranes were probed overnight at 4°C with primary antibodies such as Bax, cleaved caspase-3 (Cell Signaling Technology, 1 : 1000), and Actin (Proteintech, 1 : 1000) and then the secondary antibody (Cell Signaling Technology, 1 : 5000).

### 2.8. Serum Testosterone Analysis

About 5 mL of blood samples were taken through heart puncture and placed in biochemical tubes for the analysis of serum testosterone levels, while the rats were under deep anesthesia. After coagulation, blood was centrifuged at 4° C and 1500 g for 15 min. Serum was placed in Eppendorf tubes and kept at -80°C until analysis. The serum testosterone level of each rat was measured by ELISA according to the manufacturer's instruction (Jiancheng, Nanjing, China).

### 2.9. Computer-Aided Sperm Analyze (CASA)

The left cauda epididymis was taken, and sperm cells were extracted by a fine needle puncture. Sperm cells were then added to the prebalanced and preheated sperm capacitation fluid for 15 minutes. The capacitated sperm was diluted 100 times, and then the sperm parameter was analyzed using a computer-aided sperm analyzer (Nanning, Songjingtian Biotechnology).

### 2.10. Antioxidant Enzymes and Lipid Peroxide Analysis

Total protein concentration was determined using a protein quantification kit (Jiancheng, Nanjing, China). The protein concentration, total antioxidant capacity (T-AOC), superoxide dismutase (SOD), malondialdehyde (MDA), glutathione peroxidase (GSH-Px), glutathione (GSH), and catalase (CAT) activities or concentrations were measured according to the manufacturer's instruction (Jiancheng, Nanjing, China).

### 2.11. Transcriptome Sequencing

Total RNA was extracted and purified using an RNeasy mini kit (Qiagen, Germany). cDNA libraries were constructed using the TruSeq® Stranded Total RNA Sample Preparation Kit (Illumina, USA) following the manufacturer's instructions. Purified libraries were sequenced on an Illumina HiSeq 2000 system (Illumina, USA). Raw sequencing reads were preprocessed by filtering out rRNA reads, sequencing adapters, short fragment reads, and other low-quality reads. Tophat v2.0.9 [29] was used to map the cleaned reads to the mouse mm10 reference genome with two mismatches. After genome mapping, we ran Cufflinks v2.1.1 [30] with a reference annotation to generate FPKM values for known gene models. Fold changes were also estimated according to the FPKM in each sample. Differentially expressed genes (DEG) were selected using the following filter criteria: *P* value <0.05 and fold change <2.

### 2.12. Untargeted Metabolomic Analyses

The samples were analyzed using ultrahigh-performance liquid chromatography (1290 Infinity LC, Agilent Technologies) coupled to a quadrupole time-of-flight system (AB Sciex TripleTOF 6600). Metabolites were identified by comparing their mass spectra with an in-house database established using available authentic standards. Univariate analysis was performed for all metabolites detected between the two comparison groups (Figure S4). Subsequently, multivariate analysis was performed including principal component analysis (PCA), partial least-squares discriminant analysis (PLS-DA), and orthogonal partial least-squares discriminant analysis (OPLS-DA, Figure S5-9). The variable importance in the projection (VIP) value of each variable in the OPLS-DA model was calculated. Differential metabolites (DM) were selected using the following filter criteria: *P* value <0.05 and VIP value >1.

### 2.13. Statistical Analysis

All data were presented as mean ± SD, and statistical analysis was performed using SPSS 22.0 software (SPSS Inc., Chicago, USA). GraphPad Prism 8 Software (GraphPad Software Inc., California, USA) was used to make the graphs. Additionally, statistical significances between the two groups were determined by the Student's-*t*-test. Differences were considered statistically significant when the *P* value was less than 0.05.

## 3. Results

### 3.1. Sperm Parameter

**Figure 2:**
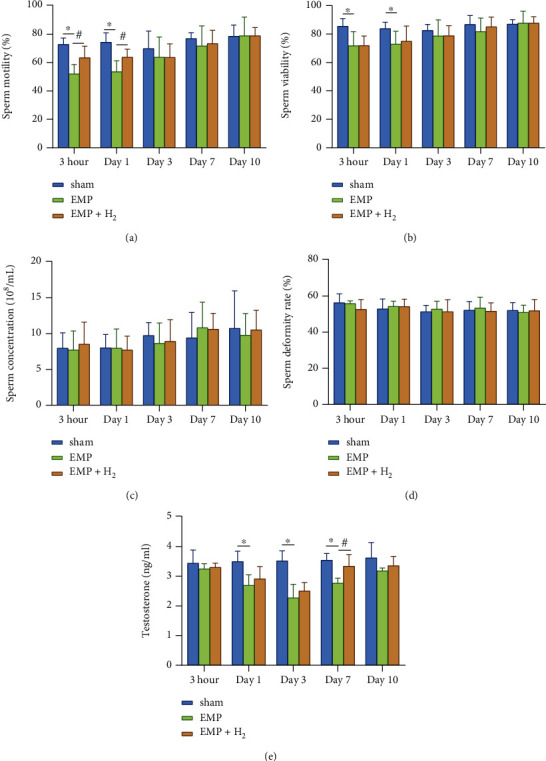
Effects of molecular hydrogen treatment on sperm quality and serum testosterone levels of rats exposed to EMP. (a) Sperm motility. (b) Sperm viability. (c) Sperm concentration. (d) Sperm deformity rate. (e) Serum testosterone levels. ∗: the difference between the EMP group and the sham group was statistically significant (*P* < 0.05). #: the difference between the EMP + H_2_ group and the EMP group was statistically significant (*P* < 0.05).

Compared with the sham group, the sperm viability and motility were decreased in the EMP group, and the results were statistically significant at 3hours and day 1 (*P* < 0.05, Figures 2(a) and 2(b)). Meanwhile, molecular hydrogen treatment significantly alleviated the decrease in sperm motility caused by exposure to EMP at 3 hours and day 1 (*P* < 0.05, Figure 2(a)). However, molecular hydrogen treatment did not show a detectable effect on sperm viability. And the sperm concentration and deformity rate seemed not to be implicated much in EMP exposure (Figures 2(c) and 2(d)).

### 3.2. Serum Testosterone Levels

Serum testosterone levels were significantly lower in the EMP group at day 1, day 3, and day 7 compared with the sham group (*P* < 0.05, Figure 2(e)). According to the line chart, the serum testosterone level reached its lowest level at day 3 and gradually recovered. Molecular hydrogen moderated the injury tendency caused by EMP exposure over the observation time course with a significant difference at day 7 (*P* < 0.05).

### 3.3. Histopathological Changes

**Figure 3:**
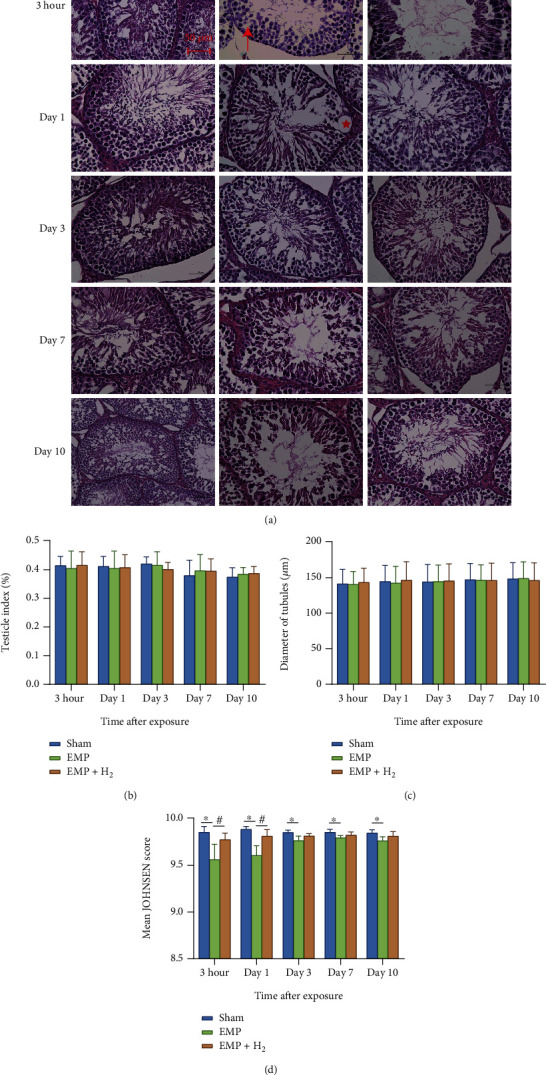
Effects of molecular hydrogen treatment on the testicular index, seminiferous tubule diameter, and Johnsen score in EMP-exposed rats. (a) Image of HE stains of testis. The red star represents the vacuole in the seminiferous tubule. The upward red arrow points to disorderly arranged spermatogenic cells. The red arrow to the left points to spermatogenic cells that separated from the basement membrane. (b) Testicular index of rats. (c) Seminiferous tubule diameter of rats measured across the minor and major axes. (d) Johnsen score of seminiferous tubules of rats. ∗: the difference between the EMP group and the sham group was statistically significant (*P* < 0.05). #: the difference between the EMP + H_2_ group and the EMP group was statistically significant (*P* < 0.05).

At different times after exposure to EMP, there was no significant change in testicular index and average testicular diameter between the sham group, the EMP group, and the EMP + H_2_ group (Figures 3(b) and 3(c)). However, compared with the sham group, the histological structure of the seminiferous tubules in the EMP group was changed. Spermatogenic cells are arranged disorderly, and even in some spermatogenic tubules, the spermatogenic cells are separated from the basement membrane, or vacuoles are common (Figure 3(a)). Compared with the sham group, testicular microstructure injuries were detected in the EMP group, which were uniformly alleviated by molecular hydrogen treatment, especially at 3 hours and day 1 (*P* < 0.05, Figure 3(d)).

### 3.4. Testicular Apoptosis

**Figure 4:**
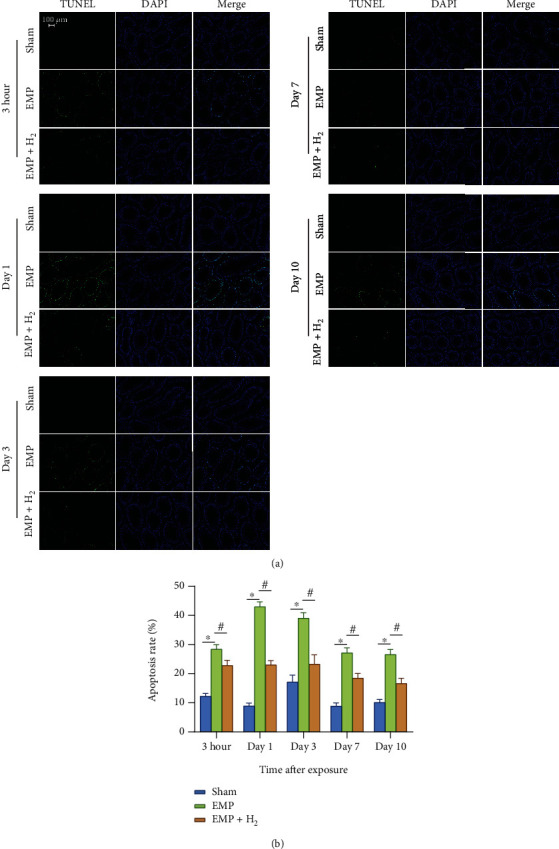
Effects of molecular hydrogen treatment on testicular cell apoptosis in rats exposed to EMP. (a) Representative images of spermatogenic cell apoptosis in testicles of rats following TUNEL staining. (b) Histogram of spermatogenic cell apoptosis rate. ∗: the difference between the EMP group and the sham group was statistically significant (*P* < 0.05). #: the difference between the EMP + H_2_ group and the EMP group was statistically significant (*P* < 0.05).

The TUNEL staining of testicular tissues is depicted in Figure 4(a). The green fluorescence in the TUNEL stains represents apoptotic cells, which were mainly distributed in the outer layer of the seminiferous tubules. This indicates that primary spermatocytes are more vulnerable to EMP. The percentages of apoptotic cells in the testis of the EMP group were significantly higher than those in the sham group and reached the highest level at day 1 (*P* < 0.05, Figure 4(b)). The percentages of apoptotic cells in the testis of the EMP + H_2_ group were significantly lower than those in the EMP group (*P* < 0.05). These data suggest that molecular hydrogen could attenuate EMP exposure-induced apoptosis in testicular cells.

**Figure 5:**
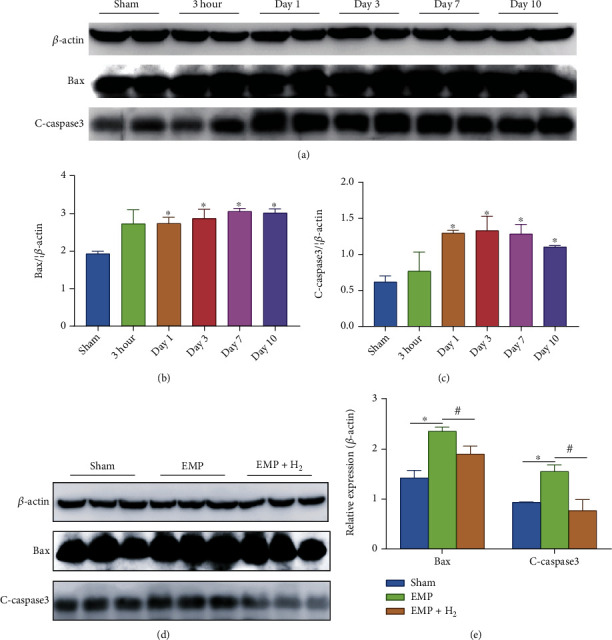
Effects of molecular hydrogen treatment on the expression levels of apoptosis proteins in the testicles of rats exposed to EMP. (a–c) Expression levels of Bax and C-caspase-3 in testis. Two samples were randomly selected from each group for western blot analysis. (d and e) Expression levels of Bax and C-caspase-3 in the testis at day 1. Three samples were randomly selected from each group for western blot analysis. ∗: the difference between the EMP group and the sham group was statistically significant (*P* < 0.05). #: the difference between the EMP + H_2_ group and the EMP group was statistically significant (*P* < 0.05).

The western blot assay of testicular tissue protein showed that molecular hydrogen could reduce the increase of apoptotic proteins induced by EMP exposure. Expression levels of Bax and cleaved caspase-3 apoptotic proteins increased in the EMP group compared to the sham group (*P* < 0.05, Figures 5(a)–5(c)). Meanwhile, molecular hydrogen could reduce the increase in the apoptotic proteins Bax and cleaved caspase-3 induced by EMP (*P* < 0.05, Figures 5(d) and 5(e)).

### 3.5. Antioxidant Enzyme, MDA, and GSH

**Figure 6:**
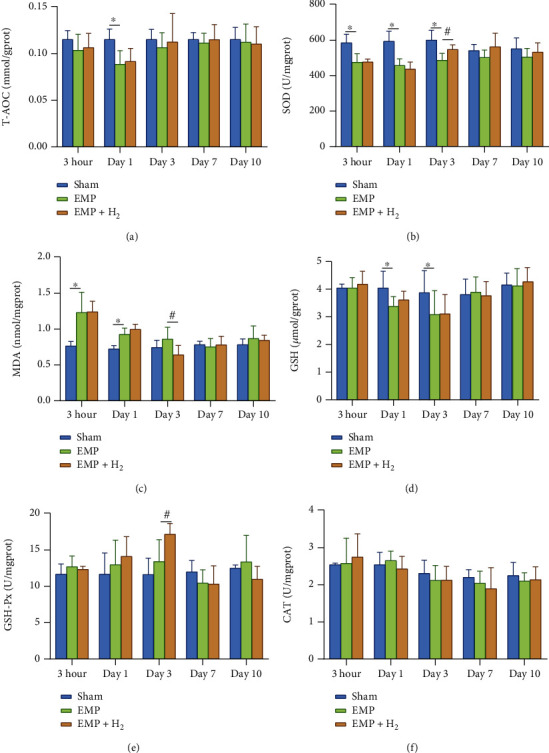
Effects of molecular hydrogen treatment on antioxidant enzymes, MDA, and GSH in the testis of rats exposed to EMP. (a) T-AOC activity. (b) SOD activity. (c) MDA levels. (d) GSH levels. (e) GSH-Px activity. (f) CAT activity. ∗: the difference between the EMP group and the sham group was statistically significant (*P* < 0.05). #: the difference between the EMP + H_2_ group and the EMP group was statistically significant (*P* < 0.05).

T-AOC activity decreased in the EMP group compared to the sham group, especially at day1. Molecular hydrogen treatment could alleviate the decrease in T-AOC activity at different time points after exposure, although the results were not statistically significant (Figure 6(a)).

The testis in the EMP group showed decreased SOD activity, especially at 3 hours, day 1, and day 3, compared to the sham group (*P* < 0.05). And molecular hydrogen treatment could alleviate the decrease of SOD activity at day 3 (*P* < 0.05, Figure 6(b)). Compared with the sham group, the MDA levels in the EMP group were increased at 3hour and day 1 (*P* < 0.05). MDA levels in the EMP + H_2_ group at day 3 were significantly lower than those in the EMP group (*P* < 0.05, Figure 6(c)). There was no significant difference in CAT activity among the sham group, the EMP group, and the EMP + H_2_ group (Figure 6(d)).

Compared with the sham group, the GSH levels in the EMP group decreased at day 1 and day 3 (*P* < 0.05). And molecular hydrogen treatment did not affect the changes of GSH levels (Figure 6(e)). However, compared with the EMP group, GSH-Px activity in the EMP + H_2_ group increased significantly at day 3 (*P* < 0.05, Figure 6(f)).

### 3.6. Identification of DEGs

Heatmap and volcano plots were used to show the changes in gene expression profiles in the H_2_ group versus the sham group, the EMP group versus the sham group, and the EMP + H_2_ group versus the EMP group (Figure S2, S3). The results showed that 106 upregulated and 113 downregulated DEGs were identified in the H_2_ group compared with the sham group. A total of 77 DEGs downregulated and upregulated, and 128 DEGs were identified in the EMP group compared to the sham group. A total of 97 upregulated and 102 downregulated DEGs were identified in the EMP + H_2_ group compared with the EMP group.

### 3.7. Gene Ontology (GO) Analyses of DEGs

**Figure 7:**
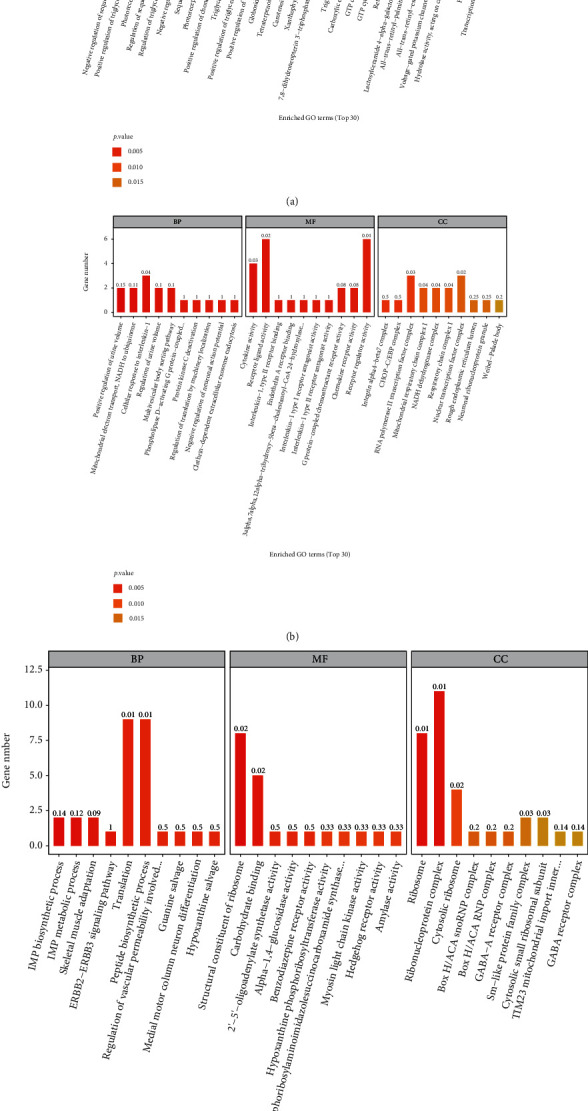
GO analyses of significant DEGs. The horizontal coordinates are the top 30 enriched GO terms under the three categories of GO (from left to right, biological processes, molecular functions, and cellular components), and the vertical coordinate is the number of DEGs annotated to the term. (a) The H_2_ group vs. the sham group. (b) The EMP group vs. the sham group. (c) The EMP + H_2_ group vs. the EMP group.

The top 30 GO terms with statistical significance for DEGs identified in the H_2_ group versus the sham group, the EMP group versus the sham group, and the EMP + H_2_ group versus the EMP group are shown in Figure 7.

### 3.8. Kyoto Encyclopedia of Genes and Genomes (KEGG) Pathway Analyses of DEGs

**Figure 8:**
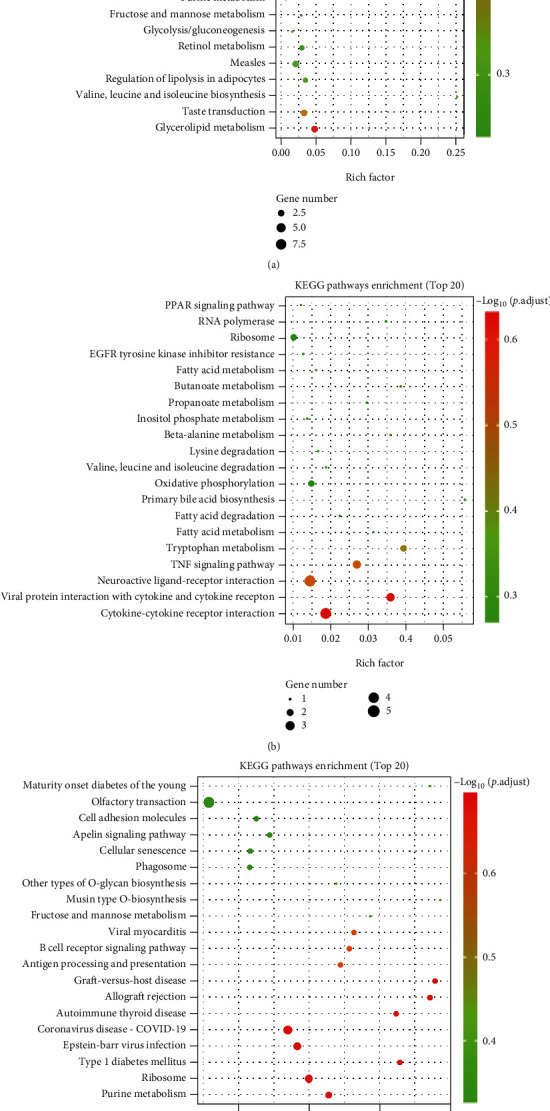
KEGG pathway analyses of DEGs identified by comparing before and after exposure to EMP or H_2_ treatment. The vertical coordinate indicates the name of the pathway, and the horizontal coordinate indicates the rich factor corresponding to the pathway. (a) The H_2_ group vs. the sham group. (b) The EMP group vs. the sham group. (c) The EMP + H_2_ group vs. the EMP group.

The molecular pathways of the identified DEGs were annotated by KEGG analysis. The results for the top 20 closely related pathways to the pathogenesis of the disease are shown in Figure 8.

### 3.9. Identification of DMs

Heatmap was used to show the changes in metabolite profiles in the H_2_ group versus the sham group, the EMP group versus the sham group, and the EMP + H_2_ group versus the EMP group.

Compared to the sham group, 16 metabolites were decreased, including DL-2-phosphoglycerate, vanillin, uracil, 5-amino-4-carbamoylimidazole, cytidine, N-acetylglucosamine-1-phosphate, arachidonic acid (free of peroxide), L-glutamate, L-threonine, 3-hydroxy-3-methyl-glutaric acid, 16-hydroxypalmitic acid, thymidine, pantothenate, citrate, isopentenyl adenosine, and methoxyacetic acid, and only benzylamine was increased in the H_2_ group.

Compared with the sham group, 12 metabolites decreased, including uric acid, adrenic acid, 2E-eicosenoic acid, myristic acid, palmitic acid, L-serine, uracil, isopentenyl adenosine, L-leucine, betaine aldehyde, L-pyroglutamic acid, and methoxyacetic acid, and 3 metabolites increased, including 3-indole propionic acid, (S)-2-hydroxyglutarate, and 2-ethyl-2-hydroxybutyric acid, in the EMP group.

**Figure 9:**
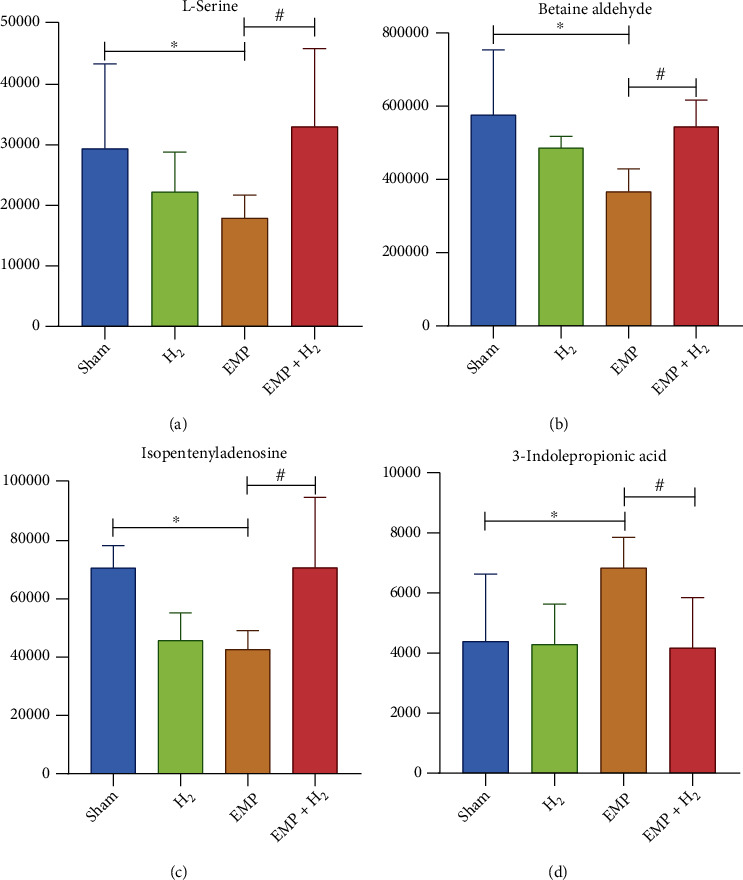
Metabolites associated with the protective effect of molecular hydrogen treatment on the testis of EMP-exposed rats. (a) L-Serine. (b) Betaine aldehyde. (c) Isopentenyladenosine. (d) 3-indolepropionic acid. ∗: the difference between the EMP group and the sham group was statistically significant (*P* < 0.05). #: the difference between the EMP + H_2_ group and the EMP group was statistically significant (*P* < 0.05).

Compared with the sham group, 16 metabolites were increased, including 3-hydroxy-3-methyl-glutaric acid, L-serine, m-chlorohippuric acid, D-ribose, L-pyroglutamic acid, betaine aldehyde, 2′-O-methylcytidine, N6-methyladenosine, xanthine, isopentenyl adenosine, betaine, pantothenate, choline, tyramine, and thymine, and 2 metabolites was decreased, including 3-indole propionic acid and allopurinol riboside in the EMP + H_2_ group. Interestingly, of the 3 increased DMs after EMP exposure, 3-indole propionic acid was decreased in the EMP + H_2_ group. Of the 12 DMs that decreased after exposure to EMP, L-serine, betaine aldehyde, and isopentenyl adenosine increased in the EMP + H_2_ group (Figure 9).

### 3.10. KEGG Pathway Analyses of DMs

**Figure 10:**
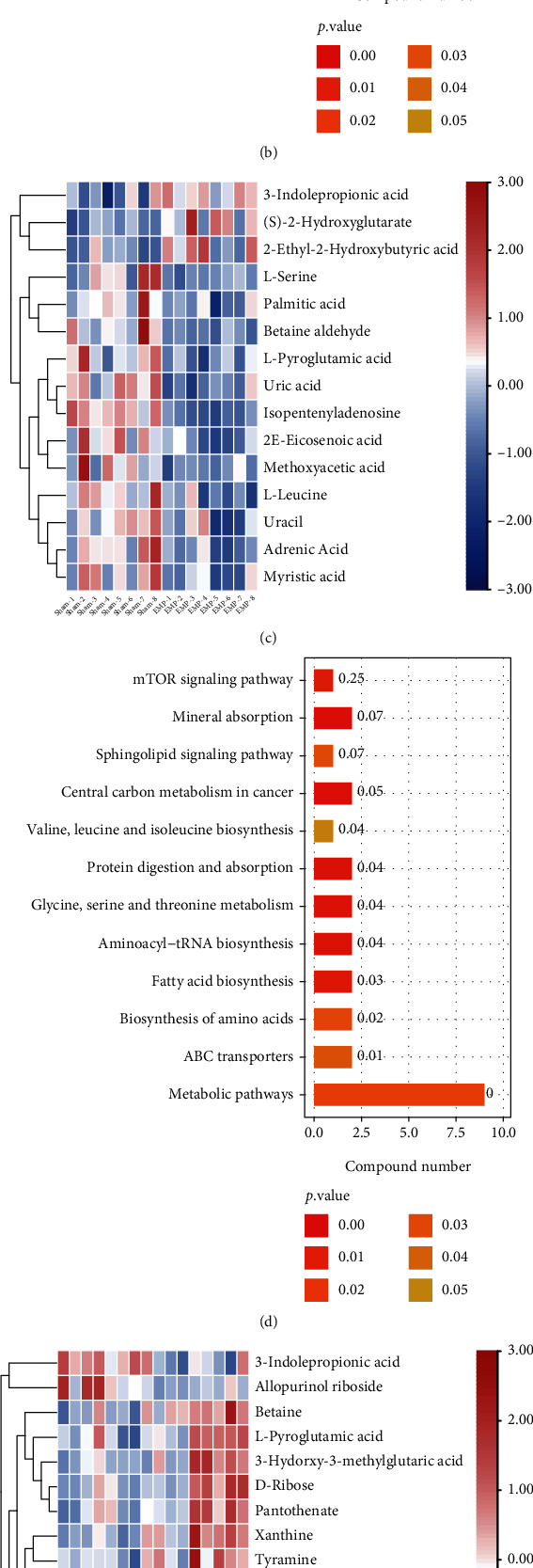
Metabolomic analysis of the protective effect of molecular hydrogen treatment on the testis of rats exposed to EMP. (a) Heatmap of DMs between the H_2_ group and the sham group. (b) KEGG pathway analysis of DMs between the H_2_ group and the sham group. (c) Heatmap of DMs between the EMP group and the sham group. (d) KEGG pathway analysis of DMs between the EMP group and the sham group. (e) Heatmap of DMs between the EMP + H_2_ and the EMP group. (f) KEGG pathway analysis of DMs between the EMP + H_2_ and the EMP group.

The molecular pathways of the identified DMs were annotated by KEGG analysis. The results for the top 20 closely related pathways to the pathogenesis of the disease are shown in Figure 10.

## 4. Discussion

While benefitting from the rapid development of communication technology, people also face the problem of the long-term and complex EMR exposure. A significant decline in sperm quality from 1940 to date has been documented in association to increased environmental pollution and changes in the lifestyle, of which EMR pollution is an important factor. It should also be taken into account that Wi-Fi-equipped mobile phones and personal computers are often located close to the reproductive organs [31]. Being reproductive functions highly sensitive to microenvironment perturbations, efforts to protect human reproductive potential are necessary and urgent.

In the present study, a rodent model was used to investigate whether molecular hydrogen could alleviate the damage to the male reproductive system caused by EMP exposure. Our data strongly support this hypothesis and suggest that molecular hydrogen treatment reduced EMP exposure-induced damage to the testis in rats. The results of CASA showed that molecular hydrogen could alleviate the decrease in sperm motility induced by EMP exposure but had no effect on sperm viability. ELISA results showed that molecular hydrogen could reduce serum testosterone decline caused by exposure to EMP and accelerate its recovery.

Sperm in the epididymis are vulnerable to oxidative stress due to lack of cytoplasmic protection and the high content of polyunsaturated fatty acids in the components of the plasma membrane of sperm. Long-term exposure to high-intensity EMR can reduce sperm concentration and motility in Cauda epididymis, thus affecting male reproductive ability. ROS are considered an important cause of EMR-induced sperm injury. Studies have found that long-term exposure to EMR increased lipid peroxidation and rupture of the middle plasma membrane in sperm [6]. The mitochondria coiled at the tail of the sperm are an important source of energy for the sperm to swim forward. A large number of studies have revealed the harmful effects of EMR from mobile phones, laptops, and other electronic devices on sperm quality and provided evidence that extensive electron leakage in the mitochondrial electron transport chain is the main cause of EMR damage [31]. Consistently, our study found that sperm viability and motility decreased significantly at 3hours and day 1, suggesting that exposure to EMP caused acute sperm injury. Molecular hydrogen treatment could significantly alleviate the decrease in sperm motility caused by exposure to EMP, which could be related to the regulation of antioxidant enzyme activity, direct reduction of ROS production, and further protection of mitochondrial function.

Oxidative stress is one of the most recognized causes of male infertility. And increasing evidence leads people to assume that EMR can interfere with the cellular oxidative/antioxidant balance, both in vitro and in vivo. There is increasing evidence suggesting that EMR exposure during spermatogenesis induces a redox imbalance due to both an increase of ROS production and a decrease in ROS scavenging activity [31]. A plethora of studies demonstrated that prolonged EMR exposure caused an increase in ROS production and an imbalance in total antioxidant capacity in terms of reduction of GSH-Px, CAT, and SOD which leads to increased lipid peroxidation in rat sperm and testis [32]. The resulting imbalance in the redox status altered the sperm cycle progression and activated the apoptotic program through the reduction of bcl-2 expression and the raise of Bax, cytochrome c, and caspase-3 protein and gene expression [31]. These findings are consistent with our results of detecting testicular apoptosis by TUNEL staining and western blot.

Molecular hydrogen can penetrate the membrane structure of cell membrane and organelle and rapidly diffuse into tissues and cells without affecting the signal transduction process. When molecular hydrogen enters the subcellular structure, it can reduce the excessive ROS and reactive nitrogen produced under pathological conditions and plays a protective role on the subcellular structure [33]. Furthermore, our study indicated that molecular hydrogen could also alleviate the decreased SOD activity, increase the GSH-Px activity, and finally increase the T-AOC in organisms, exerting its protective effect on EMP exposure. SOD is an antioxidant metal enzyme present in organisms. It can catalyze superoxide anion radical disproportionation to produce oxygen and hydrogen peroxide, which plays a vital role in the balance of oxidation and antioxidation in vivo. GSH-Px is another important peroxidase enzyme that exists widely in vivo. GSH-Px promotes the reaction of hydrogen peroxide with reduced glutathione (GSH) to produce water and oxidize glutathione. Our results indicated that EMP exposure affected the balance of antioxidant enzyme systems in the testis of rats, resulting in decreased T-AOC and SOD activities. EMP exposure led to inefficient and delayed clearance of excess free radicals in the testis, which significantly increased MDA levels. The experimental results showed that molecular hydrogen treatment could increase antioxidant enzyme activity and reduce MDA levels. This may be the mechanism by which molecular hydrogen plays a role in alleviating the damage of exposure to EMP to the male reproductive system.

It has been experimentally demonstrated that molecular hydrogen could selectively scavenge hydroxyl radicals and nitro peroxide anions [18]. However, the specific mechanism by which molecular hydrogen functions in living organisms has not been elucidated until now. To further explore the mechanism of molecular hydrogen in protecting rat testis from EMP exposure, transcriptomic and untargeted metabolomics were used to search for potential genes, metabolites, or pathways.

Currently, some research reports [34, 35] have conducted beneficial investigations, but we are the first to utilize transcriptomic and untargeted metabolomics to analyze the mechanism of EMP exposure and molecular hydrogen on rat testis. The present study indirectly reflected the difference in gene expression by comparing the difference in gene transcript abundance between the H_2_ group and the sham group and found 106 upregulated and 113 downregulated DEGs, strongly suggesting that molecular hydrogen can cause significant changes in the gene expression profile of rat testis. Through GO analyses of DEGs, it was realized that molecular hydrogen mainly affects biological processes such as triglyceride metabolism, as well as some essential molecular functions such as triglyceride lipase activity and carboxylate hydrolase activity. KEGG pathway analyses of DEGs revealed that molecular hydrogen mainly affects amino acid biosynthesis, metabolic pathways, retinol metabolism, adipocytes lipolysis, taste conduction, and glycerolipid metabolism. Furthermore, KEGG pathway analyses of DMs revealed that molecular hydrogen mainly influences central carbon metabolism in cancer, central carbon metabolism in cancer, pyrimidine metabolism, amino acid biosynthesis, carbon metabolism, and ABC transporters. On the one hand, our findings deepened the understanding that molecular hydrogen could scavenge the hydroxyl radical and affect intracellular metabolism [36]. However, it is still urgently needed to further clarify the biological mechanism underlying molecular hydrogen.

In the present study, 77 up regulated and 128 downregulated DEGs were identified by comparing the EMP group and the sham group. GO analyses of DEGs revealed that EMP mainly affects biological processes such as positive regulation of urine volume, mitochondrial electron transport (NADH to ubiquinone), and cellular response to IL-1, as well as molecular functions such as cytokine activity and receptor-ligand activity. KEGG pathway analyses of DEGs revealed that EMP mainly affects ribosomes, oxidative phosphorylation, TNF signaling pathway, and cytokine-cytokine receptor interaction. KEGG pathway analyses of DMs revealed that EMP mainly affects metabolic pathways such as fatty acid biosynthesis, amino tRNA biosynthesis, and (Gly, serine, and threonine) metabolism and signaling pathways such as the mTOR signaling pathway and sphingolipid signaling pathway.

Compared with the EMP group, the EMP + H_2_ group had 97 upregulated and 102 downregulated DEGs. GO analyzes revealed that DEGs in the EMP + H_2_ group versus the EMP group were mainly enriched in molecular functions such as structural components of the ribosome and carbohydrate binding, cellular components such as the ribosome, the ribonucleoprotein complex, and the cytoplasmic ribosome. KEGG pathway analyses showed that DEGs in the EMP + H_2_ group versus the EMP group were mainly enriched in olfactory conduction, cell adhesion molecules, Apelin signaling pathway, cellular senescence, and phagosome. KEGG pathway analyses of DMs revealed that EMP mainly affects (Gly, serine, and threonine) the metabolism, the sphingolipid signaling pathway, and the ABC transporters. Interestingly, 3-indole propionic acid was increased in the EMP group (vs. the sham group) and decreased in the EMP + H_2_ group (vs. the EMP group). Meanwhile, L-serine, betaine aldehyde, and isopentenyl adenosine have been decreased in the EMP group (vs. the sham group) and increased in the EMP + H_2_ group (vs. the EMP group). From the above results, we believe that the protective mechanism of molecular hydrogen is related to its effect on intracellular ribosome, cell adhesion molecules, cellular senescence, and phagosome. The protective mechanism of molecular hydrogen is also related to its reversal of metabolite changes caused by EMP exposure.

Molecular hydrogen, as a kind of diatomic molecule with weak reducibility, has anti-inflammatory and antioxidant effects in many animal and clinical experiments [26, 37–39]. At present, molecular hydrogen has been found to have certain therapeutic effects on more than 200 diseases, and similar results have been achieved in preliminary clinical trials. Interestingly, anaerobic bacteria in the human intestinal tract continuously produce hydrogen during anaerobic fermentation every day. The reported amount of hydrogen generated per day is between 150 mL and 12 L [40]. Kajiya et al. [41] found that antibacterial drugs eliminated hydrogen-producing bacteria in the mouse intestinal tract and aggravated drug-induced hepatitis. These results indicated that the hydrogen produced by anaerobic bacteria plays an essential role in the physiological maintenance of organisms.

In many studies, it has been found that EMP exerts a detrimental effect on reproductive system including testis, leading to decreased sperm quality [42, 43]. Also, many researchers have been trying to explore methods to prevent these damages, for example, some of whom have observed that melatonin or some other natural extracts can protect against these injuries [9, 15–17]. Recently, it has been reported that molecular hydrogen combined with Korean red ginseng extract can improve spermatogenesis and sperm motility in male mice [44]. In the current study, however, it is first time for molecular hydrogen to be reported to play a protective role against acute sperm damage caused by EMP exposure, which is in accordance with our previous work that molecular hydrogen can alleviate the ionizing radiation injuries by selectively scavenging reactive oxygen species [20, 22, 45, 46]. Moreover, our findings are also consistent with those of others that molecular hydrogen can also mitigate oxidative damages induced by other factors such as hydrogen peroxide, greatly expanding its potential application scope [47].

As a potential EMR protection agent, molecular hydrogen has many advantages, such as convenient use, small toxic and side effects, and good curative effect. However, the research on molecular hydrogen is still in the preclinical stage, and the clinical research using molecular hydrogen is still rare, requiring large-scale clinical research. We are currently conducting a clinical intervention research on the treatment of male infertility with molecular hydrogen in our Reproductive Medicine Center, in the hope of providing us with more clinical support. Until now, the specific mechanism by which molecular hydrogen functions in living organisms has not been elucidated. Although experimental evidence such as molecular hydrogen could selectively inhibit hydroxyl free radicals and peroxynitrite anion has been obtained, the specific metabolic pathways of hydrogen in vivo are still unclear.

## 5. Conclusion

Our study found that molecular hydrogen can reduce the damage of EMP exposure to the reproductive system of male rats, and its protective mechanism related to intracellular ribosome, cell adhesion molecules, cellular senescence, phagosome, and intracellular metabolism. Molecular hydrogen might be used as a new strategy to prevent electromagnetic radiation-induced damage on the male reproductive system.

## Data Availability

All data used to support the findings of this study were provided by the Naval Medical University and cannot be freely available. Requests for access to these data should be made to the corresponding author on a reasonable request.

## Supplementary Materials

Supplementary Materials Figure S1: decline curve of hydrogen concentration in freshly prepared hydrogen-rich water after exposure to air. The dotted line represents the saturation concentration of hydrogen. And when the temperature is 26°C, the salt concentration is 0‰, and the saturation concentration of hydrogen at standard atmospheric pressure is 776.79 (*μ*mol/L). Figure S2: volcano plot of the differential gene expression profile. Each point in the differential expression volcano plot represents a gene, the abscissa represents the logarithm of the difference in the expression level of a gene between two samples, and the ordinate represents the negative logarithm of the *P* value. The green dots represent downregulated DEGs, the red dots represent upregulated DEGs, and the black dots represent non-DEGs. (A) The H_2_ group vs. the sham group. (B) The EMP group vs. the sham group. (C) The EMP+ H_2_ vs. the EMP group. Figure S3: heatmap of the differential expression profile of genes. Each column represents one sample, and each row represents one gene. Red indicates upregulation and blue indicates downregulation. The upper side shows the tree diagram of the sample clustering. The closer the branches of two samples are to each other, the closer the expression pattern of all differential genes in these two samples. The left side shows the tree diagram of gene clustering. The closer the two gene branches are to each other, the closer their expressions are. (A) The H_2_ group vs. the sham group. (B) The EMP group vs. the sham group. (C) The EMP+ H_2_ vs. the EMP group. Figure S4 Volcano plot of the differential expression profile of metabolites. The horizontal coordinate is the log2 value of the differential expression multiplier, and the vertical coordinate is the log10 value of the significant *P* value. Metabolites meeting FC>1.5 and *P* value <0.05 are shown in red, and metabolites that meet FC<0.67 and *P* value <0.05 are shown in blue. Nonsignificantly different metabolites are shown in black. (A) The H_2_ group vs. the sham group (positive and negative ion mode). (B) The EMP group vs. the sham group (positive and negative ion mode). (C) The EMP vs. the EMP+ H_2_ group (positive and negative ion mode). Figure S5: PCA score graph of metabolites. In the figure, *t*[1] represents principal component 1, *t*[2] represents principal component 2, and the ellipse represents the 95% confidence interval. The dots of the same color indicate the individual biological replicates within the group. The distribution status of the points reflects the degree of variation between and within the groups. (A) The H_2_ group vs. the sham group (positive and negative ion mode). (B) The EMP group vs. the sham group (positive and negative ion mode). (C) The EMP+ H_2_ vs. the EMP group (positive and negative ion mode). Figure S6: PLS-DA score graph of metabolites. In the figure, *t*[1] represents principal component 1, *t*[2] represents principal component 2, and the ellipse represents the 95% confidence interval. The dots of the same color indicate the individual biological replicates within the group. The distribution status of the points reflects the degree of variation between and within groups. (A) The H_2_ group vs. the sham group (positive and negative ion mode). (B) The EMP group vs. the sham group (positive and negative ion mode). (C) The EMP+ H_2_ vs. the EMP group (positive and negative ion mode). Figure S7: PLS-DA permutation test of metabolites. The horizontal coordinate indicates the replacement retention, i.e., the proportion that is consistent with the order of the original model *Y* variables, and the vertical coordinate indicates the *R*^2^ and *Q*^2^ values. The green dots indicate *R*^2^, the blue dots indicate *Q*^2^, and the two dashed lines indicate the regression lines of *R*^2^ and *Q*^2^, respectively. The *R*^2^ and *Q*^2^ in the upper right corner indicate that the replacement retention is equal to 1, i.e., the *R*^2^ and *Q*^2^ values of the original model. (A) The H_2_ group vs. the sham group (positive and negative ion mode). (B) The EMP group vs. the sham group (positive and negative ion mode). (C) The EMP+ H_2_ vs. the EMP group (positive and negative ion mode). Figure S8: OPLS-DA score graph of metabolites. In the figure, *t*[1] represents principal component 1, *t*[2] represents principal component 2, and the ellipse represents the 95% confidence interval. The dots of the same color indicate the individual biological replicates within the group. The distribution status of the points reflects the degree of variation between and within groups. (A) The H_2_ group vs. the sham group (positive and negative ion mode). (B) The EMP group vs. the sham group (positive and negative ion mode). (C) The EMP+ H_2_ vs. the EMP group (positive and negative ion mode). Figure S9: OPLS-DA permutation test of metabolites. The horizontal coordinate indicates the replacement retention, i.e., the proportion that is consistent with the order of the original model *Y* variables, and the vertical coordinate indicates the *R*^2^ and *Q*^2^ values. The green dots indicate *R*^2^, the blue dots indicate *Q*^2^, and the two dashed lines indicate the regression lines of *R*^2^ and *Q*^2^, respectively. The *R*^2^ and *Q*^2^ in the upper right corner indicate that the replacement retention is equal to 1, i.e., the *R*^2^ and *Q*^2^ values of the original model. (A) The H_2_ group vs. the sham group (positive and negative ion mode). (B) The EMP group vs. the sham group (positive and negative ion mode). (C) The EMP+ H_2_ vs. the EMP group (positive and negative ion mode).
