# Supplementary material for: Molecular Hydrogen Reduces Electromagnetic Pulse-Induced Male Rat Reproductive System Damage in a Rodent Model
Source: Oxid Med Cell Longev. 2022 Sep 25;2022:3469474. doi: 10.1155/2022/3469474 (PMC9527415; doi:10.1155/2022/3469474)
Supplement: Supplementary Materials — Figure S1: decline curve of hydrogen concentration in freshly prepared hydrogen-rich water after exposure to air. The dotted line represents the saturation concentration of hydrogen. And when the temperature is 26°C, the salt concentration is 0‰, and the saturation concentration of hydrogen at standard atmospheric pressure is 776.79 (μmol/L). Figure S2: volcano plot of the differential gene expression profile. Each point in the differential expression volcano plot represents a gene, the abscissa represents the logarithm of the difference in the expression level of a gene between two samples, and the ordinate represents the negative logarithm of the P value. The green dots represent downregulated DEGs, the red dots represent upregulated DEGs, and the black dots represent non-DEGs. (A) The H2 group vs. the sham group. (B) The EMP group vs. the sham group. (C) The EMP+ H2 vs. the EMP group. Figure S3: heatmap of the differential expression profile of genes. Each column represents one sample, and each row represents one gene. Red indicates upregulation and blue indicates downregulation. The upper side shows the tree diagram of the sample clustering. The closer the branches of two samples are to each other, the closer the expression pattern of all differential genes in these two samples. The left side shows the tree diagram of gene clustering. The closer the two gene branches are to each other, the closer their expressions are. (A) The H2 group vs. the sham group. (B) The EMP group vs. the sham group. (C) The EMP+ H2 vs. the EMP group. Figure S4 Volcano plot of the differential expression profile of metabolites. The horizontal coordinate is the log2 value of the differential expression multiplier, and the vertical coordinate is the log10 value of the significant P value. Metabolites meeting FC>1.5 and P value <0.05 are shown in red, and metabolites that meet FC<0.67 and P value <0.05 are shown in blue. Nonsignificantly different metabolites are shown in black. (A) The H2 [file 3469474.f1.docx]

**Figure S1** Decline curve of hydrogen concentration in freshly prepared hydrogen-rich water after exposure to air. The dotted line represents the saturation concentration of hydrogen. And when the temperature is 26℃, the salt concentration is 0‰, the saturation concentration of hydrogen at standard atmospheric pressure is 776.79 (µmol/L).


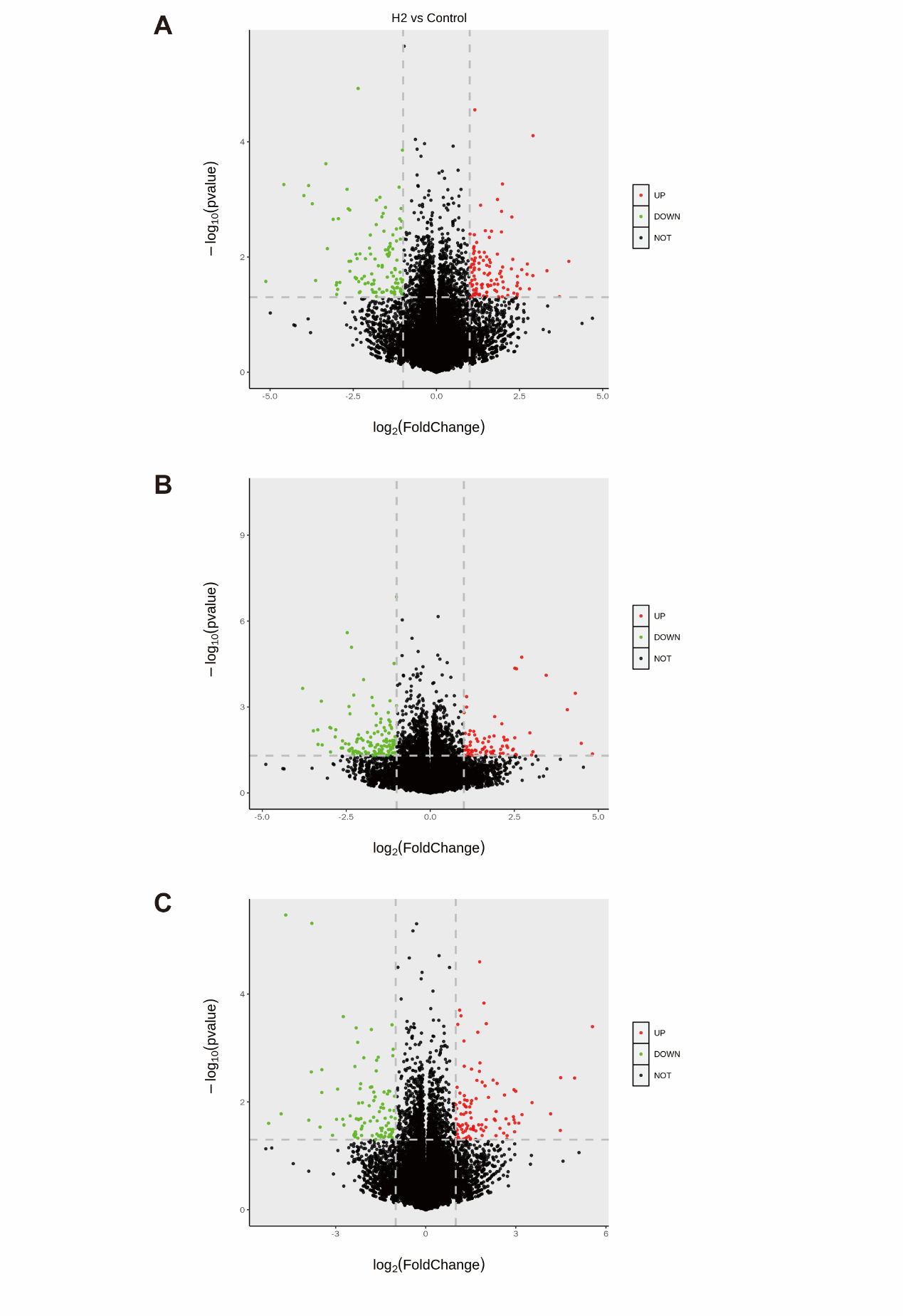


**Figure S2** Volcano plot of the differential gene expression profile. Each point in the differential expression volcano plot represents a gene, the abscissa represents the logarithm of the difference in the expression level of a gene between two samples, and the ordinate represents the negative logarithm of the P-value. The green dots represent down-regulated DEGs, the red dots represent up-regulated DEGs, and the black dots represent non-DEGs. (**A**) The H_2_ group vs. the sham group. (**B**) The EMP group vs. the sham group. (**C**) The EMP+ H_2_ vs. the EMP group.


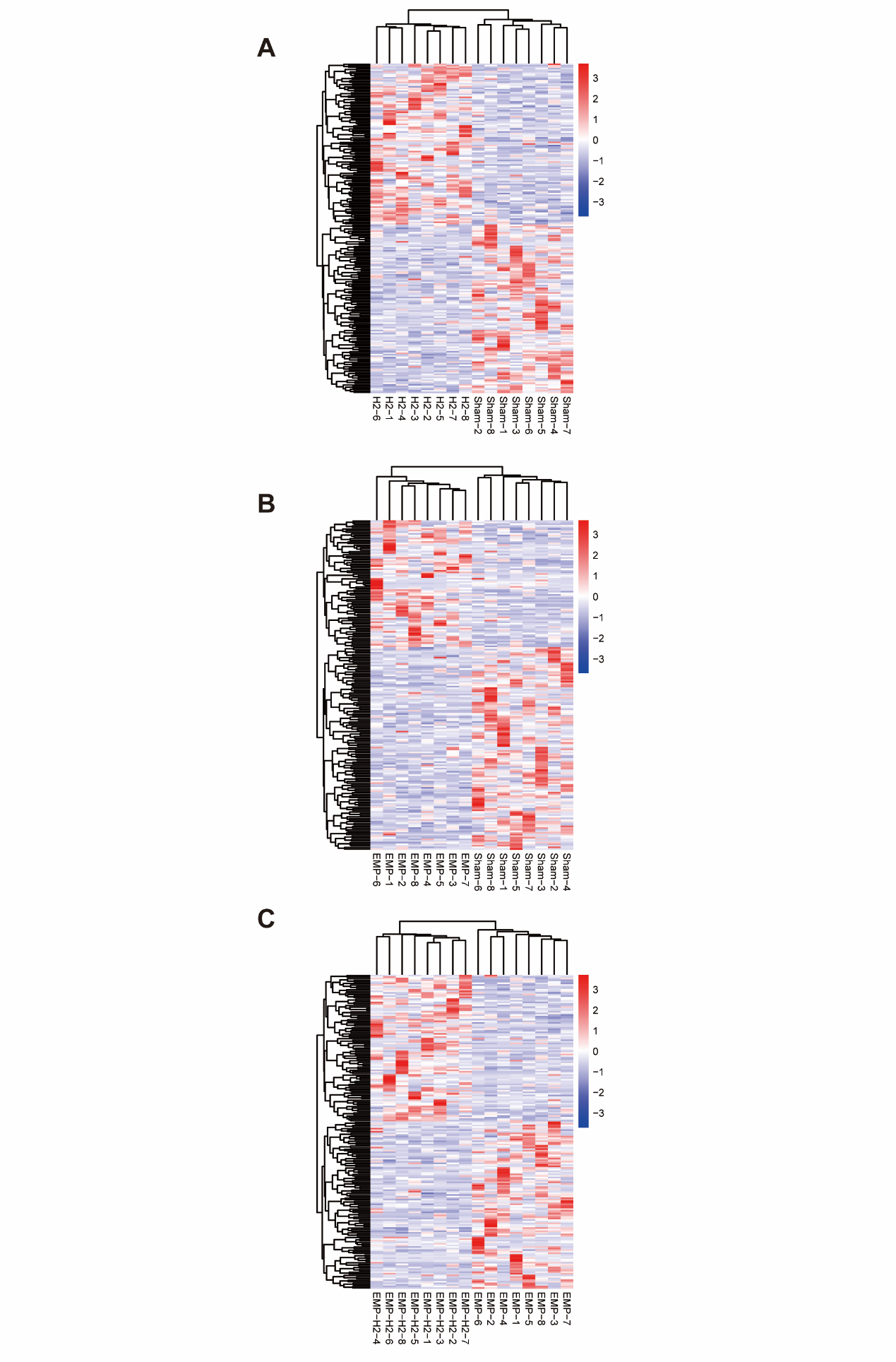


**Figure S3** Heatmap of the differential expression profile of genes. Each column represents one sample, and each row represents one gene. Red indicates up-regulation and blue indicates down-regulation. The upper side shows the tree diagram of the sample clustering. The closer the branches of two samples are to each other, the closer the expression pattern of all differential genes in these two samples. The left side shows the tree diagram of gene clustering. The closer the two gene branches are to each other, the closer their expressions are. (**A**) The H_2_ group vs. the sham group. (**B**) The EMP group vs. the sham group. (**C**) The EMP+ H_2_ vs. the EMP group.


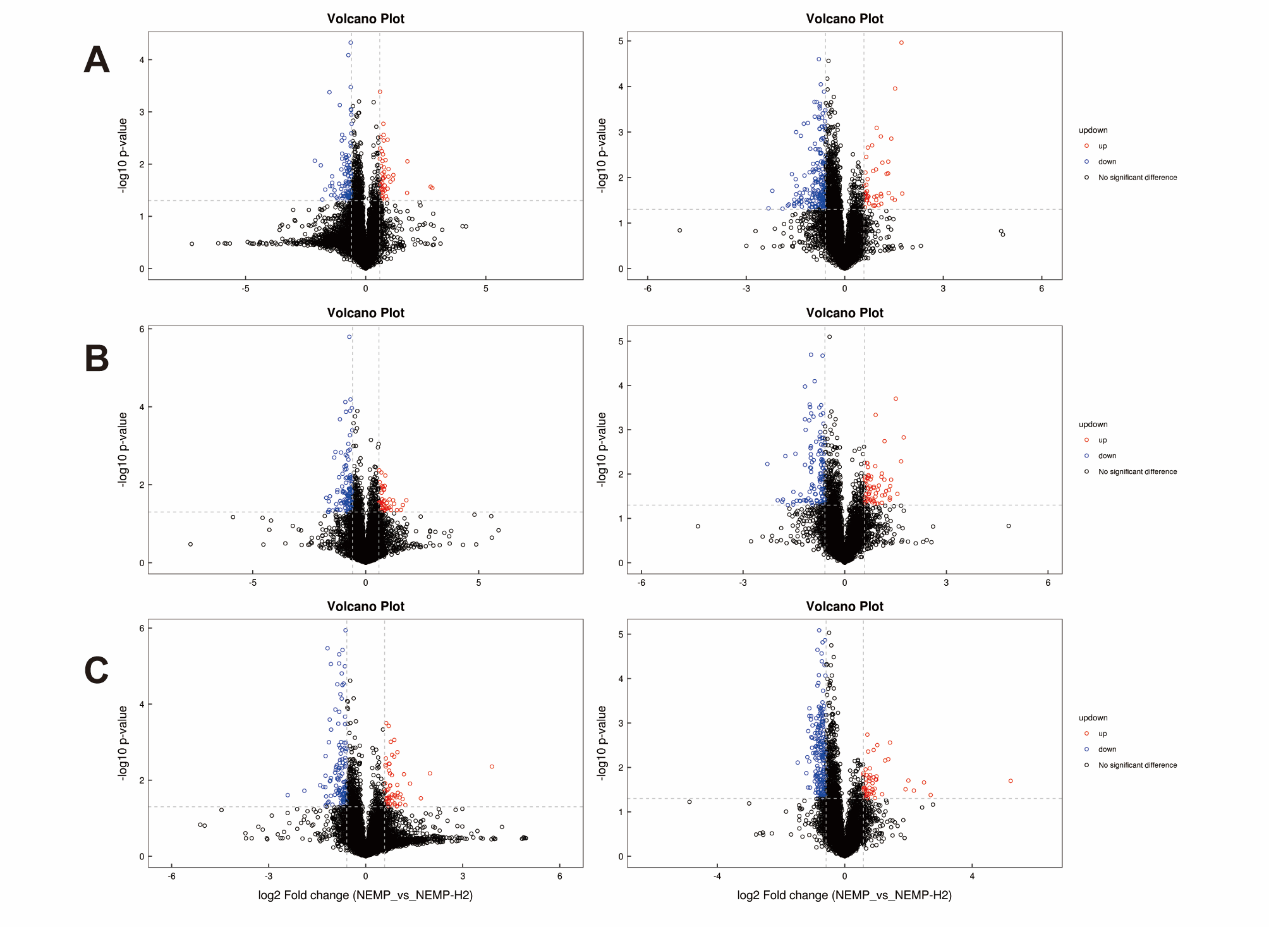


**Figure S4** Volcano plot of the differential expression profile of metabolites. The horizontal coordinate is the log2 value of the differential expression multiplier, and the vertical coordinate is the log10 value of the significant P-value. Metabolites meeting FC > 1.5 and P-value < 0.05 are shown in red, and metabolites that meet FC < 0.67 and P-value < 0.05 are shown in blue. Non-significantly different metabolites are shown in black. (**A**) The H_2_ group vs. the sham group (positive and negative ion mode). (**B**) The EMP group vs. the sham group (positive and negative ion mode). (**C**) The EMP vs. the EMP+ H_2_ group (positive and negative ion mode).


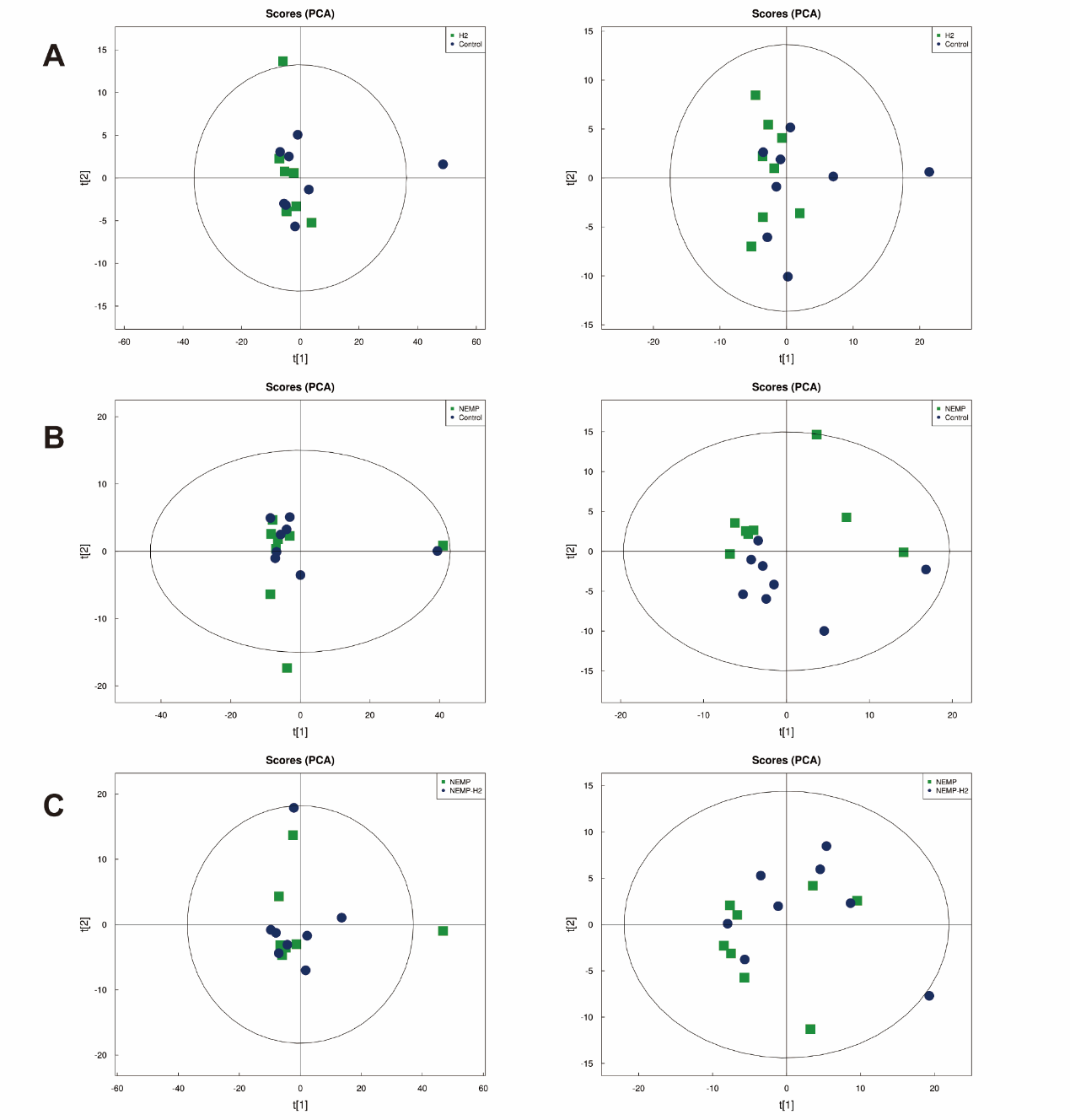
**Figure S5** PCA score graph of metabolites. In the figure, t[1] represents principal component 1, t[2] represents principal component 2, and the ellipse represents the 95% confidence interval. The dots of the same color indicate the individual biological replicates within the group. The distribution status of the points reflects the degree of variation between and within the groups. (**A**) The H_2_ group vs. the sham group (positive and negative ion mode). (**B**) The EMP group vs. the sham group (positive and negative ion mode). (**C**) The EMP+ H_2_ vs. the EMP group (positive and negative ion mode).


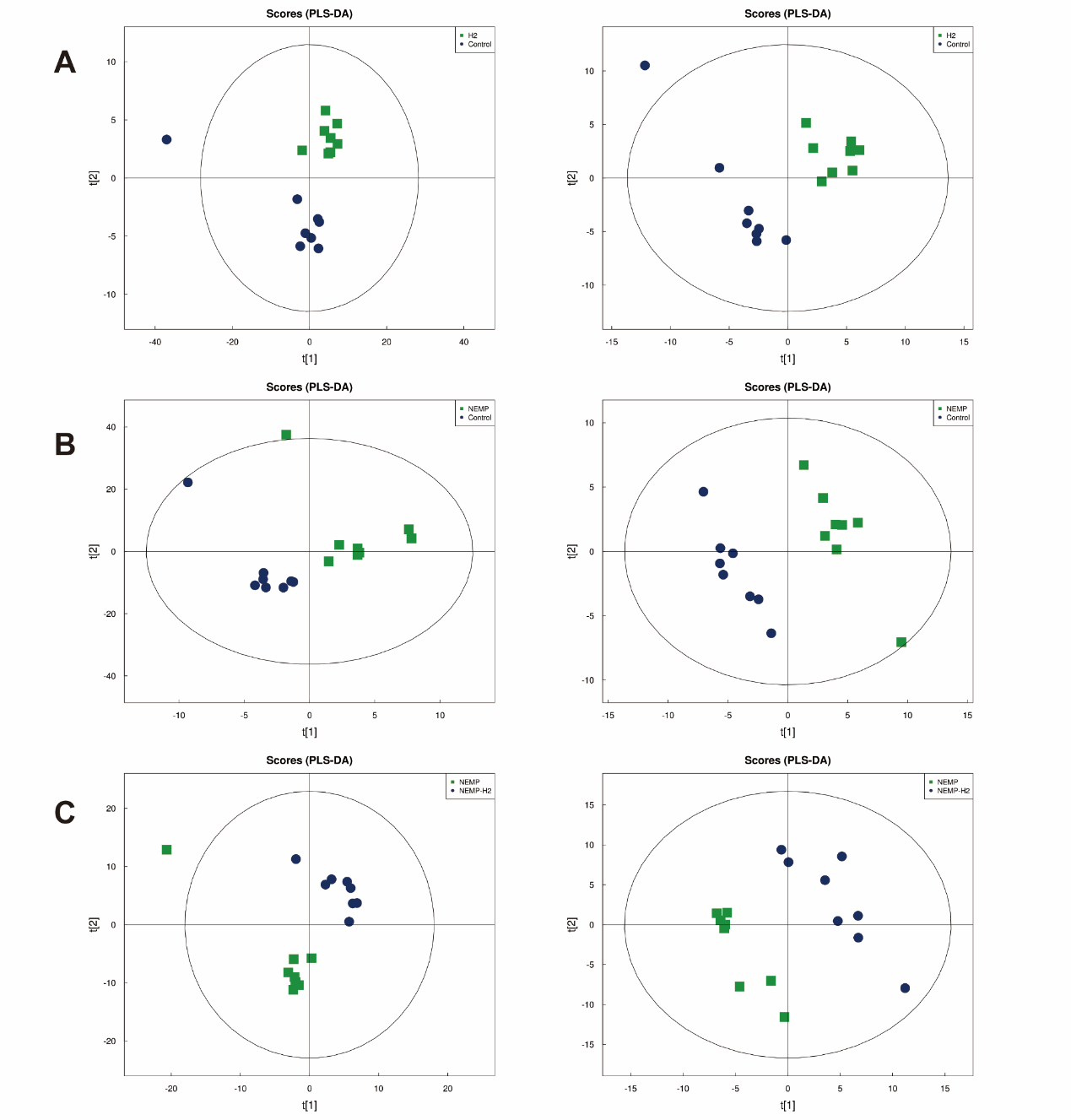
**Figure S6** PLS-DA score graph of metabolites. In the figure, t[1] represents principal component 1, t[2] represents principal component 2, and the ellipse represents the 95% confidence interval. The dots of the same color indicate the individual biological replicates within the group. The distribution status of the points reflects the degree of variation between and within groups. (**A**) The H_2_ group vs. the sham group (positive and negative ion mode). (**B**) The EMP group vs. the sham group (positive and negative ion mode). (**C**) The EMP+ H_2_ vs. the EMP group (positive and negative ion mode).


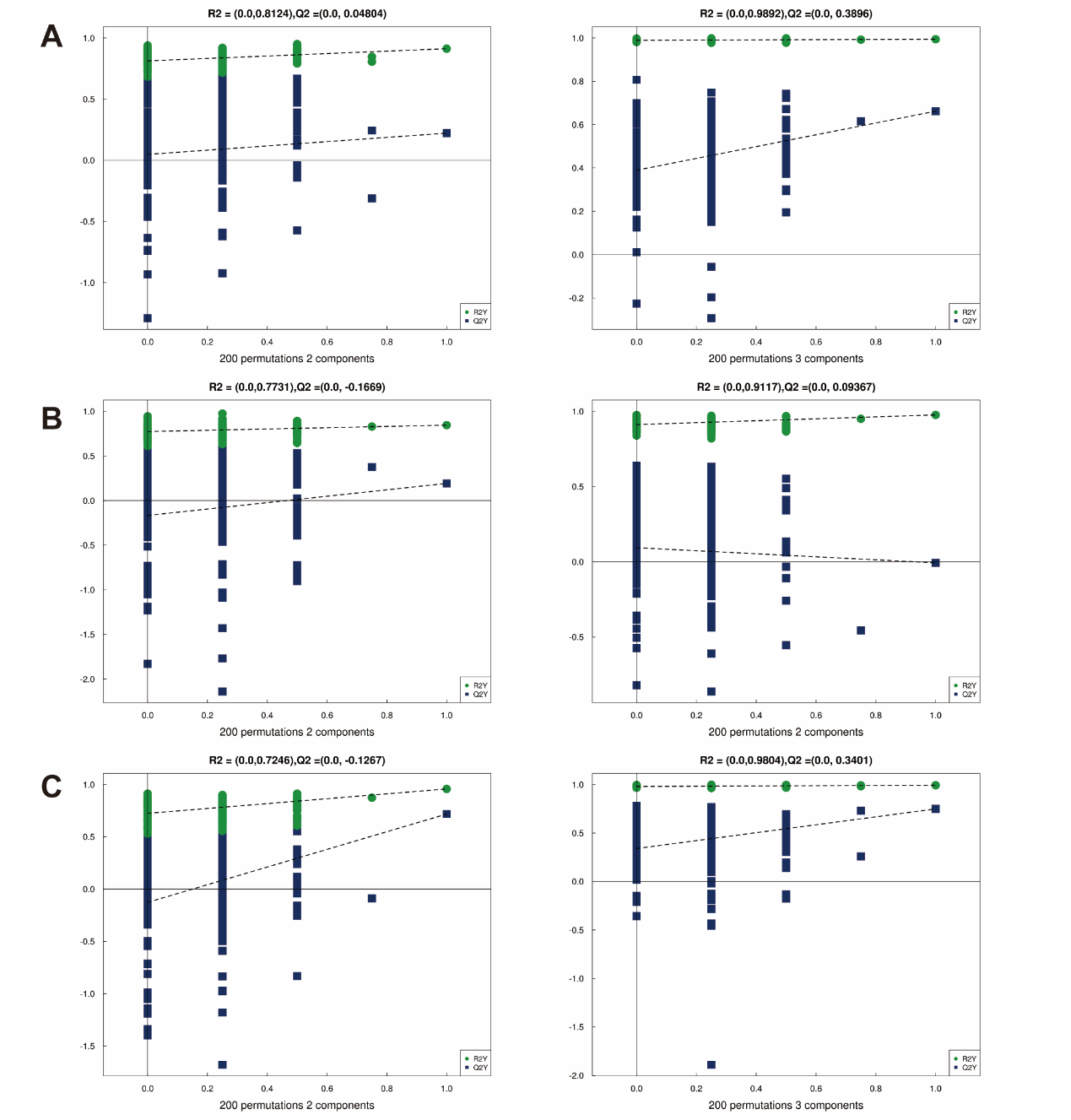


**Figure S7** PLS-DA permutation test of metabolites. The horizontal coordinate indicates the replacement retention, i.e., the proportion that is consistent with the order of the original model Y variables, and the vertical coordinate indicates the R^2^ and Q^2^ values. The green dots indicate R^2^, the blue dots indicate Q^2^, and the two dashed lines indicate the regression lines of R^2^ and Q^2^, respectively. The R^2^ and Q^2^ in the upper right corner indicate that the replacement retention is equal to 1, i.e., the R^2^ and Q^2^ values of the original model. (**A**) The H_2_ group vs. the sham group (positive and negative ion mode). (**B**) The EMP group vs. the sham group (positive and negative ion mode). (**C**) The EMP+ H_2_ vs. the EMP group (positive and negative ion mode).


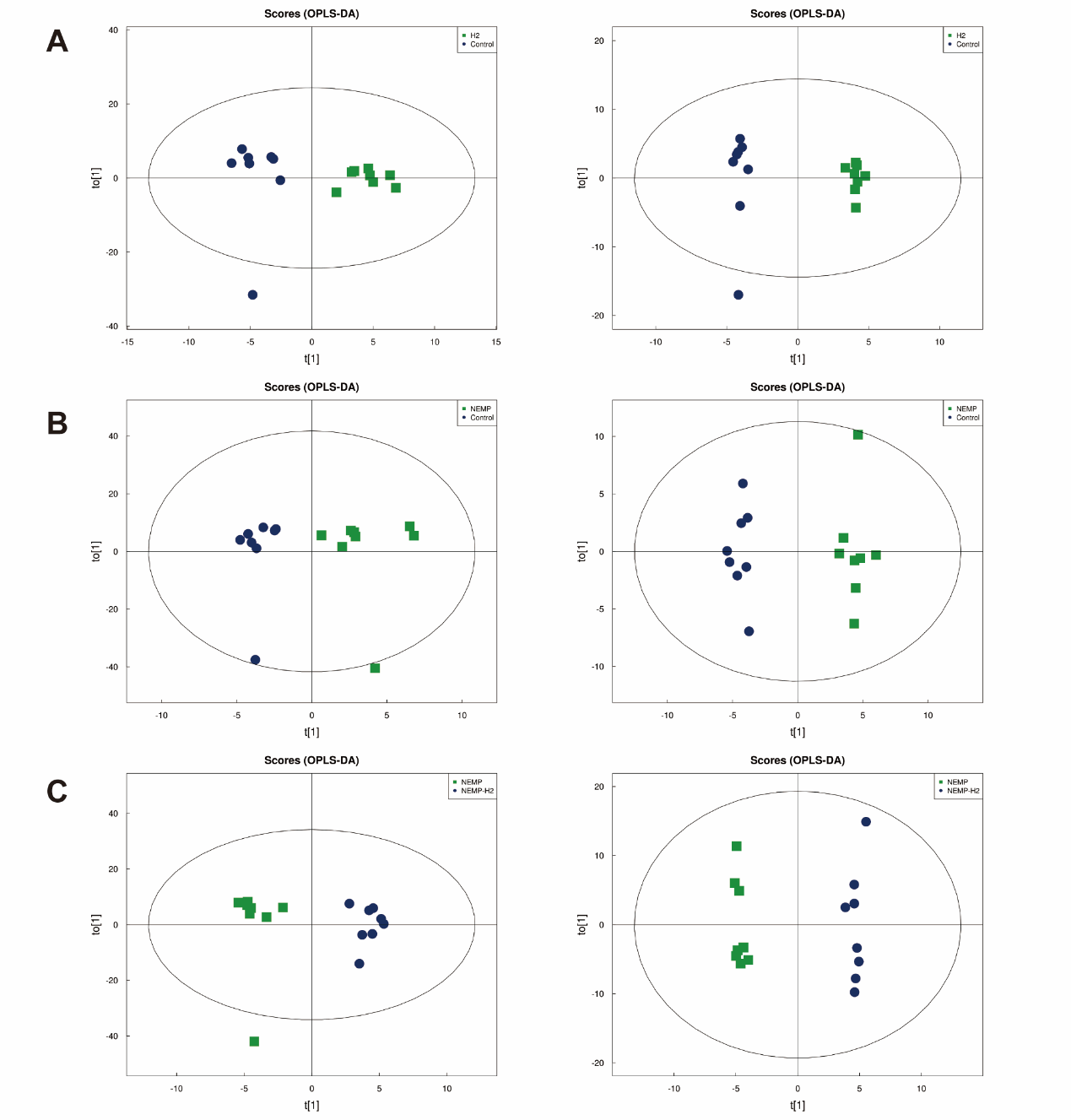
 **Figure S8** OPLS-DA score graph of metabolites. In the figure, t[1] represents principal component 1, t[2] represents principal component 2, and the ellipse represents the 95% confidence interval. The dots of the same color indicate the individual biological replicates within the group. The distribution status of the points reflects the degree of variation between and within groups. (**A**) The H_2_ group vs. the sham group (positive and negative ion mode). (**B**) The EMP group vs. the sham group (positive and negative ion mode). (**C**) The EMP+ H_2_ vs. the EMP group (positive and negative ion mode).


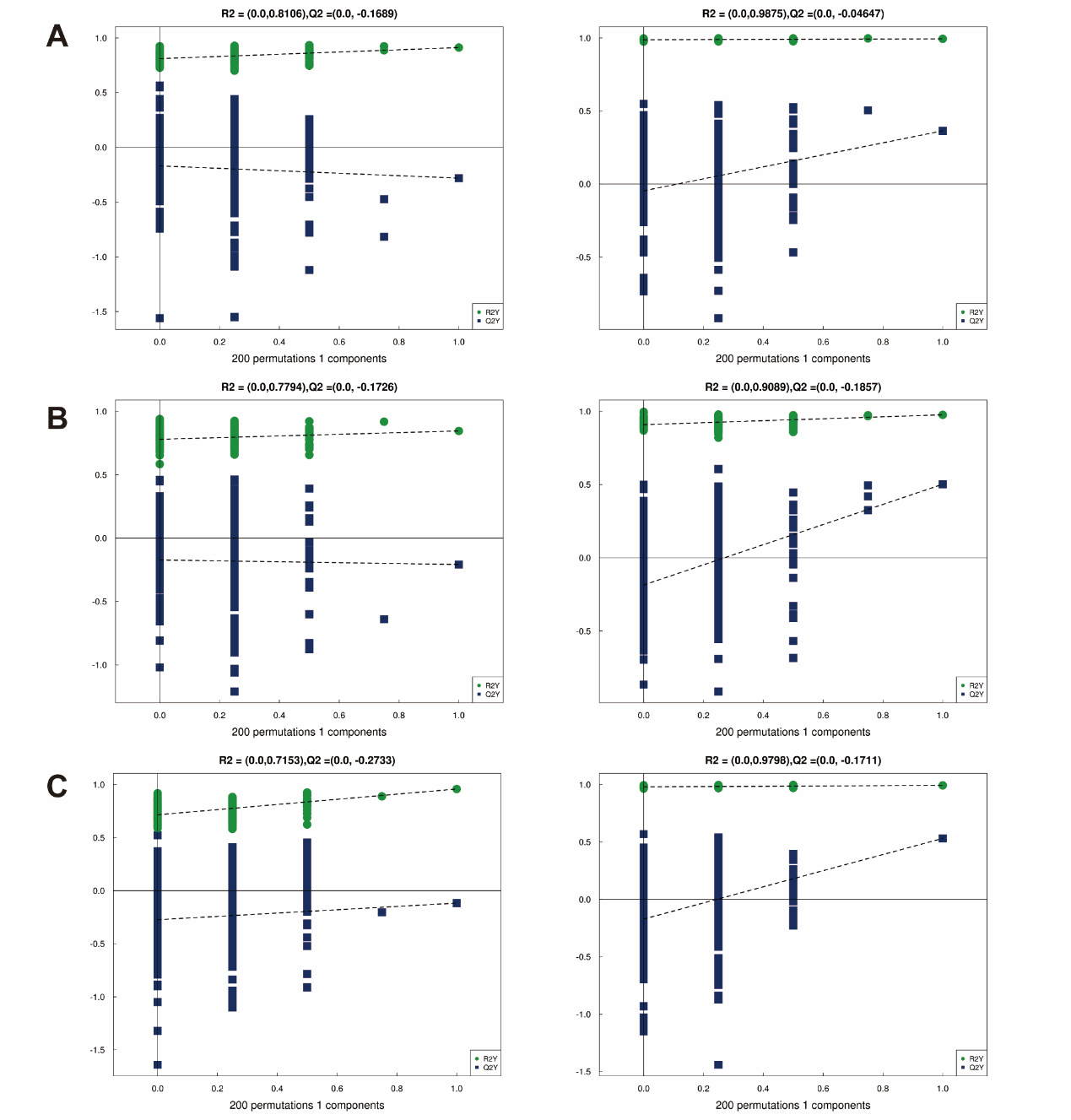


**Figure S9** OPLS-DA permutation test of metabolites. The horizontal coordinate indicates the replacement retention, i.e., the proportion that is consistent with the order of the original model Y variables, and the vertical coordinate indicates the R^2^ and Q^2^ values. The green dots indicate R^2^, the blue dots indicate Q^2^, and the two dashed lines indicate the regression lines of R^2^ and Q^2^, respectively. The R^2^ and Q^2^ in the upper right corner indicate that the replacement retention is equal to 1, i.e., the R^2^ and Q^2^ values of the original model. (**A**) The H_2_ group vs. the sham group (positive and negative ion mode). (**B**) The EMP group vs. the sham group (positive and negative ion mode). (**C**) The EMP+ H_2_ vs. the EMP group (positive and negative ion mode).
